# Platelet aging and desialylation increase apoptotic priming and BCL-X_L_ dependence

**DOI:** 10.1038/s41419-025-08205-8

**Published:** 2026-01-08

**Authors:** Renata Grozovsky, Cameron S. Fraser, Xingping Qin, Johan Spetz, Kristopher A. Sarosiek

**Affiliations:** 1 Miller School of Medicine, University of Miami, Miami, FL USA; 2 Molecular and Integrative Physiological Sciences Program, Harvard T.H. Chan School of Public Health, Boston, MA USA; 3 John B. Little Center for Radiation Sciences, Harvard T.H. Chan School of Public Health, Boston, MA USA; 4 Laboratory of Systems Pharmacology, Harvard Program in Therapeutic Science, Department of Systems Biology, Harvard Medical School, Boston, MA USA; 5 Department of Medical Oncology, Dana-Farber Cancer Institute, Boston, MA USA

**Keywords:** Translational research, Apoptosis, Targeted therapies

## Abstract

Platelets are short-lived anucleate cells essential for primary hemostasis and recognized for their functions in thrombosis, immunity, antimicrobial defense, neurodegeneration, as well as cancer growth and metastasis. Their brief lifespan in circulation is controlled by the removal of sialic acid residues from the platelet surface (desialylation) and also the mitochondrial apoptosis pathway, with high expression of the anti-apoptotic protein BCL-X_L_ being required for platelet survival. This dependence on BCL-X_L_ has prevented the clinical deployment of recently developed small molecule inhibitors of BCL-X_L_, which have promising activity in solid as well as liquid cancers but cause on-target thrombocytopenia. Here, we investigate the functional relationship between platelet desialylation and apoptosis to determine how cross-talk between these mechanisms may impact platelet lifespan. We find that platelets progressively lose sialic acid residues and become more primed for apoptosis while in circulation, resulting in aged platelets that are desialylated and highly prone to undergoing apoptosis. In addition, platelet desialylation via endogenous or exogenous factors directly increases their BCL-X_L_ dependence and accelerates apoptosis, which can be reversed by treatment with the sialidase inhibitor DANA (2,3-dehydro-2-deoxy-N-acetylneuraminic acid). Notably, young platelets recently released into circulation are less primed for apoptosis and less dependent on BCL-X_L_ for survival. Consistent with these changes in priming, platelets aged in vitro exhibit increasing expression of multiple pro-apoptotic proteins including BIM, BAK and PUMA along with increasing cleaved caspase 3. Leveraging the lower BCL-X_L_ dependence of young platelets, stimulation of de novo platelet production with the thrombopoietin receptor agonist romiplostim prevents BH3 mimetic-induced thrombocytopenia in vivo and may prevent severe platelet loss in patients treated with BCL-X_L_ inhibitors.

## Introduction

Platelets are small, anucleate cells produced by megakaryocytes primarily in the bone marrow and then released into circulation where they survive for 4–5 days in mice and 7–10 days in humans [1]. Platelets are critical for primary hemostasis, where their function is to rapidly form clots at sites of injury to avoid bleeding [2]. Aside from the control of bleeding and clotting, platelets also play important roles in inflammation, atherosclerosis, host defense, and tumor metastasis, acting as sentinels and responding to environmental changes through their cell surface receptors [3–7]. Therefore, maintenance of the platelet population number and function is central to regulate tissue homeostasis. In humans and mice, platelets are produced and cleared from circulation daily. Dysregulated platelet clearance can have life-threatening consequences such as thrombocytopenia (low platelet counts), one of the major causes of morbidity in clinical disorders such as sepsis or cancer [5, 8].

Similar to nucleated cells, platelets carry an array of surface glycans important for maintaining protein stability, enabling cell-cell interactions and ligand-receptor affinity [9]. Sialic acid is a key determinant of platelet survival as loss of sialic acid from the platelet surface (desialylation) exposes galactose as a terminal residue and leads to the recognition and rapid clearance of platelets from circulation by the hepatic asialoglycoprotein Ashwell–Morell receptor (AMR) [10]. This work also demonstrated that platelet desialylation is a naturally occurring platelet aging process but more importantly, it showed that clearance of desialylated platelets by the AMR triggers the JAK2-STAT3 signaling pathway and increases thrombopoietin and platelet production [11]. Sialic acid (or Neuraminic acid) residues are removed from the platelet surface by the action of sialidases or neuraminidases (NEU1-4) [12]. Neu1 is expressed in all mammalian tissues including platelets, where can be recruited to the cell surface [12]. The compound 2,3-dehydro-2-deoxy-N-acetylneuraminic acid (DANA) is a broad-spectrum neuraminidase inhibitor of all four NEU isozymes [13, 14].

In addition to clearance by desialylation, platelet levels are also regulated by apoptosis [15, 16]. Apoptosis is a regulated form of cell death that is tightly controlled by changes in protein expression, protein-protein interactions, and various post-translational modifications of BCL-2 family proteins [17, 18]. As anucleate cells, platelets lack some of the upstream stress and damage signaling pathways that can induce mitochondrial (intrinsic) apoptosis (e.g., DNA damage, cell cycle checkpoint violation) [19, 20]. Still, studies using genetically modified mice observed that knock out mice for pro-apoptotic effector proteins BAK and BAX have prolonged platelet survival [21]. Moreover, pharmacological inhibition of pro-survival proteins BCL-2, BCL-X_L_ and BCL-w using ABT-737 [22] and ABT-263 [23] (navitoclax) accelerated platelet clearance, demonstrating that the presence of the intrinsic apoptotic machinery plays a role on platelet lifespan. Platelet survival was then thought to be dependent on a balance between pro-survival and pro-apoptotic family of proteins; however, we and others have found unchanged levels of BCL-X_L_ in aged platelets [16]. Furthermore, in vivo studies using BAD knockout mice showed only a modest improvement in platelet survival while knockout of BID, BIM or PUMA had no impact [24]. The physiological trigger that initiates platelet apoptosis remains unknown.

In recent years, BH3 mimetics such as ABT-263 and ABT-737, which inhibit activity of pro-survival proteins BCL-2, BCL-X_L_ and BCL-W, have proven to be highly effective at eliminating cancer cells by directly inducing apoptosis [25–27]. Many BH3 mimetics are well-tolerated, but BH3 mimetics targeting BCL-X_L_ have stalled in clinical development due to on-target thrombocytopenia [28].

In this study, we show that as platelets age and become desialylated in circulation and in vitro, they become more primed to undergo apoptosis and increase their dependence on BCL-X_L_. Notably, preventing desialylation with the neuraminidase inhibitor, DANA, or stimulating de novo production of platelets reduces BCL-X_L_ inhibitor-induced platelet loss both in vitro and in vivo. By identifying the signaling mechanisms triggering platelets apoptosis, our findings enable the development of agents that specifically promote platelet survival by maintaining platelet sialylation to potentially treat life-threatening thrombocytopenia and thromboses.

## Results

### Aged platelets become desialylated and undergo caspase-mediated apoptosis

**Fig. 1:**
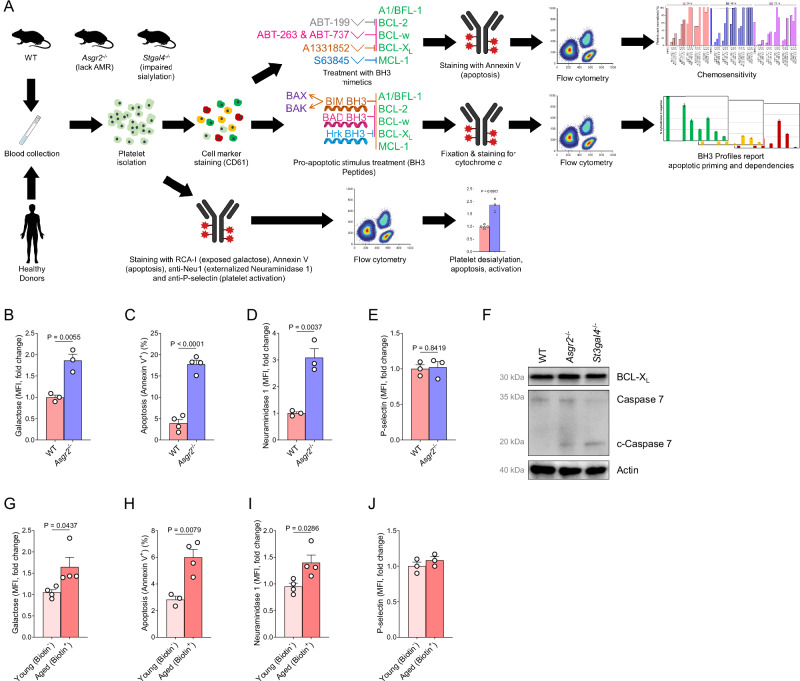
Desialylated platelets are prone to undergoing apoptosis. **A** Workflow of platelet collection and processing for BH3 profiling, chemosensitivity and protein expression analysis. Flow cytometry analysis of WT (control) and desialylated platelets derived from *Asgr2*^–/–^ mice using **B** RCA-I lectin to detect exposed galactose, **C** Annexin V to detect apoptosis, **D** anti-Neu1 antibody to detect surface expression of Neu1 and **E** anti-P-selectin antibody to measure activation. Mean ± SEM is shown for *n* = 3-4 biological replicates, as indicated by individual data points. **F** WT and *Asgr2*^–/–^ platelet lysates were subjected to immunoblotting with antibodies recognizing BCL-X_L_, full length and cleaved caspase 7, or actin as loading control. Data are representative of *n* = 3 biological replicates. Flow cytometry analysis of young (biotin^−^) and aged (biotin^+^) platelet populations after magnetic bead sorting using **G** RCA-I lectin to detect exposed galactose, **H** Annexin V to detect apoptosis, **I** anti-Neu1 antibody to detect surface expression of Neu1 and **J** anti-P-selectin antibody to measure activation. Mean ± SEM is shown for *n* = 3-4 biological replicates, as indicated by individual data points.

We and others have shown that desialylated platelets are rapidly cleared from circulation by the hepatic AMR [10, 11]. In *Asgr2*^–/^^–^ mice, which lack subunit 2 of the AMR, clearance of desialylated platelets is impaired and they accumulate in circulation [11]. To test whether platelet desialylation and aging was also linked to the induction of apoptosis, we analyzed platelets freshly collected from several strains of mice that have altered platelet clearance (Fig. 1A). We first confirmed increased galactose exposure, which results from sialic acid removal, on the platelet surface in *Asgr2*^–/–^ mice by staining cells with the lectin Ricinus Communis Agglutinin I (RCA-I), which specifically binds galactose [29] (Fig. 1B). We next measured platelet apoptosis by staining with Annexin V to detect exposure of phosphatidylserine (PS), an indicator of apoptotic cell death. Desialylated platelets derived from *Asgr2*^*-/-*^ mice were found to have increased levels of PS exposure (Fig. 1C), indicating higher rates of ongoing apoptosis. We also detected higher Neuraminidase 1 (Neu 1) surface exposure on *Asgr2*^–/–^ platelets compared to WT (Fig. 1D), which are presumably contributing to platelet desialylation. It is important to note that surface expression of P-selectin, a marker of platelet activation [30], is not increased in *Asgr2*^–/–^ platelets (Fig. 1E), indicating that platelet desialylation or aging does not affect their activation status. We also found that platelets from *Asgr2*^–/–^ mice expressed higher levels of cleaved caspase 7 (Fig. 1F), which is in agreement with the increased Annexin V staining and indicates that aged platelets are undergoing higher rates of apoptosis.

We next used in vivo biotinylation to evaluate the relationship of platelet aging and apoptosis in WT mice. After intravenous injection, biotin covalently labels free amino groups on the surface of all cells in circulation. Platelets produced after biotin administration will remain biotin negative, allowing for direct comparison of aged (biotin^+^) and young (biotin^−^) platelets (Fig. S1A, B). We found that aged (biotin^+^) platelets exhibited increased galactose exposure as shown by RCA-I binding compared to young (biotin^−^) platelets, confirming increased desialylation (Fig. 1G). Similar to platelets derived from *Asgr2*^–/–^ mice, aged (biotin^+^) platelets exhibited higher levels of surface PS and Neu1 exposure when compared to young (biotin^−^) platelets (Fig. 1H, I). Platelet integrity and activation were unaffected by biotinylation as determined by cellular granularity (Fig. S1C) and staining for P-selectin [11] (Fig. 1J).

### Platelet apoptosis does not induce desialylation

**Fig. 2:**
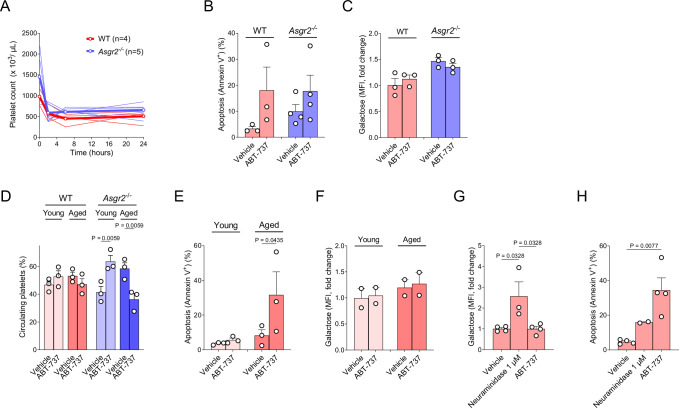
ABT-737 treatment induces platelet apoptosis without causing desialylation. **A** Blood platelet count of WT and *Asgr2*^–/–^ mice following a single intraperitoneal injection of ABT-737. Data collected from each mouse as well as mean is shown for *n* = 4–5 mice/group. Flow cytometry analysis of platelets collected from mice 90 min after ABT-737 injection using **B** Annexin V to detect apoptosis and **C** RCA-I lectin to detect exposed galactose. Mean ± SEM is shown for *n* = 3–4 biological replicates, as indicated by individual data points. **D** Percentage of circulating platelets: young (biotin^−^) and aged (biotin^+^) platelet populations in WT and *Asgr2*^–/–^ mice 90 min after a single intraperitoneal injection of ABT-737 using biotinylation to compare young versus aged. Mean ± SEM is shown for *n* = 3 biological replicates. Flow cytometry analysis of platelets derived from WT mice after biotin (60 h) and ABT-737 (90 min) injections: **E** Annexin V to detect apoptosis, and **F** RCA-I lectin to detect exposed galactose. Mean ± SEM is shown for *n* = 2–3 biological replicates. Flow cytometry analysis of freshly washed WT platelets treated with neuraminidase (1 μM) or ABT-737 (5 μM) for 20 min in vitro at 37 °C using **G** RCA-I to detect exposed galactose and **H** Annexin V to detect apoptosis. Mean ± SEM is shown for *n* = 2–4 biological replicates, as indicated by individual data points.

We next sought to investigate if triggering apoptosis affected platelet sialylation status. First, WT and *Asgr2*^–/–^ mice received a single dose of the BH3 mimetic ABT-737, which inhibits BCL-2, BCL-X_L_ and BCL-W and has been previously reported to induce platelet apoptosis [22]. Platelet count was measured at 0, 2, 6 and 24 h after ABT-737 injection, and we observed a significant decrease in platelet counts in both WT and *Asgr2*^–/–^ mice 2 h after injection of ABT-737 (Fig. 2A). Although the *Asgr2*^–/–^ mice started with higher platelet counts, the number of platelets after administration of ABT-737 was virtually identical in both sets of mice, indicating that older, desialylated platelets are cleared more efficiently from circulation after BH3 mimetic administration.

We also measured PS and galactose exposure by Annexin V and RCA-I binding, respectively, 90 min after ABT-737 injection, a time point shortly before platelets are cleared from circulation and found that ABT-737 induced platelet apoptosis in both WT and *Asgr2*^–/–^ mice (Fig. 2B) but did not promote desialylation (Fig. 2C), indicating that platelets were undergoing apoptosis without changing their sialylation status.

Next, we injected a single dose of biotin in WT and *Asgr2*^–/–^ mice and, 48 h later, mice received a single injection of ABT-737. We again collected blood after 90 min and measured PS exposure using Annexin V staining. We found that ABT-737 treatment caused greater loss of aged platelets from circulation in both WT and *Asgr2*^–/–^ mice (Fig. 2D). We also detected increased levels of ABT-737-induced apoptosis in aged platelets compared with young (Fig. 2E) but no effect on desialylation (Fig. 2F).

To complement our in vivo data, we treated freshly collected and washed WT platelets in vitro with neuraminidase at doses previously shown to directly desialylate platelets [31] or ABT-737. Neuraminidase treatment induced platelet desialylation (Fig. 2G) and, notably, also triggered apoptosis (Fig. 2H). Conversely, ABT-737 treatment induced a high rate of apoptosis (Fig. 2H) but did not induce desialylation, as evidenced by unaltered RCA-I binding (Fig. 2G). Collectively, these data indicate that direct induction of apoptosis does not cause desialylation, while desialylation, endogenous or pharmacological, induces platelet apoptosis.

### Desialylated and aged platelets are more primed for apoptosis

Cellular commitment to mitochondrial apoptosis is regulated by the BCL-2 family of proteins [17]. To gain insight into the regulation of apoptosis in platelets, we utilized the BH3 profiling assay, which measures apoptotic priming (proximity to the threshold at which a cell commits to apoptosis) as well as dependence on pro-survival BCL-2 family proteins [32, 33]. In BH3 profiling, cells are gently permeabilized and treated with pro-apoptotic BH3 peptides, which mimic the activity of full-length BH3-only proteins and come in two classes: (1) activator BH3 peptides (BIM and BID) that can inhibit all pro-survival proteins and also directly activate the effector proteins BAX and BAK; and (2) sensitizer BH3 peptides (BAD, HRK, MS1) that can only inhibit specific pro-survival proteins to indicate dependence on these proteins for survival. After a 60-min peptide treatment, mitochondrial cytochrome c release, which is an indicator of mitochondrial outer membrane permeabilization (MOMP) – the key commitment step in mitochondrial apoptosis – is measured using antibody staining. Cytochrome c release in response to activator peptides or the PUMA peptide, which inhibit all pro-survival proteins, provides a measure of overall apoptotic priming. Cytochrome c release in response to peptides that inhibit specific pro-survival proteins indicates dependence on them for survival. BH3 profiling has been previously used to measure apoptotic priming and dependencies in normal cells [20, 34–37] as well as cancers [38, 39].

**Fig. 3:**
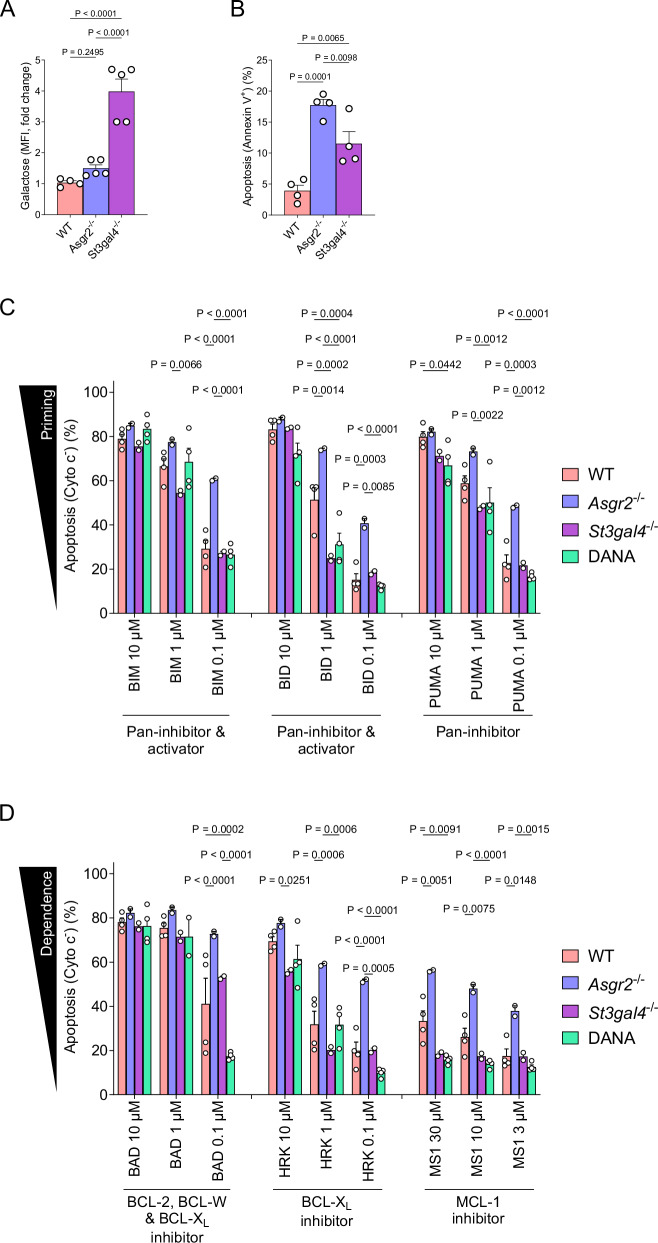
Desialylated platelets are more primed for apoptosis and dependent on BCL-X_L_. Flow cytometry analysis of freshly isolated platelets collected from WT, *Asgr2*^–/–^ and *St3gal4*^–/–^ mice using **A** RCA-I to detect exposed galactose and **B** Annexin V to detect apoptosis. Mean ± SEM is shown for *n* = 4–5 biological replicates, as indicated by individual data points. BH3 profiling of freshly isolated platelets derived from WT, *Asgr2*^–/–^ and *ST3Gal4*^–/–^ or WT mice treated with 2,3-dehydro-2-deoxy-N-acetylneuraminic acid (DANA) for 48 h using **C** BIM and BID BH3 activator peptides that inhibit all pro-survival proteins and also directly activate BAX and BAK and the PUMA BH3 sensitizer peptide that only inhibits all pro-survival proteins but does not activate BAX or BAK and **D** BAD BH3, HRK and MS1 peptides that only inhibit indicated pro-survival proteins and do not activate BAX or BAK. Cytochrome c release indicates initiation of apoptosis and indicates level of apoptotic priming or dependence on pro-survival proteins. Mean ± SEM is shown for *n* = 2–4 biological replicates, as indicated by individual data points.

To investigate the effect desialylation has on platelet apoptotic sensitivity, platelets from WT, *Asgr2*^–/–^, and *St3gal4*^–/–^ mice were isolated and subjected to BH3 profiling using BH3 peptides and doses that have been previously shown to be potent and selective [32, 33, 40]. The sialyltransferase ST3Gal-IV adds sialic acid onto terminal galactose residues on the platelet surface [41–43]. Consequently, platelets derived from *St3gal4*^–/–^ mice are rapidly cleared by the hepatic AMR and those in circulation are highly desialylated but chronologically young [44], which we confirmed by detecting four-fold higher RCA-I binding in platelets from these mice compared to WT (Fig. 3A). Interestingly, these platelets were also undergoing higher rates of apoptosis in vivo as measured by Annexin V binding (Fig. 3B) as well as increased levels of cleaved caspase 7 (Fig. 1F). These data, along with the endogenous or forced desialylation described above, indicate that lack of sialylation is a pro-apoptotic stressor to platelets.

BH3 profiling analysis demonstrated that WT platelets are primed for apoptosis, as indicated by their proficient release of cytochrome c from mitochondrial in response to BIM, BID and PUMA BH3 peptides at even relatively low doses (Fig. 3C). Platelets from *Asgr2*^–/–^ mice released higher amount of cytochrome c in response to the BIM and BID BH3 peptides when compared to WT platelets, indicating that aged, desialylated platelets are more primed for apoptosis (Fig. 3C). Interestingly, platelets from *St3gal4*^–/–^ mice exhibited reduced sensitivity to the BID BH3 peptide at the 1 μM dose, indicating that young, desialylated platelets surviving in circulation are slightly less primed for apoptosis (Fig. 3C).

### Desialylated and aged platelets exhibit increased dependence on BCL-X_L_

We next tested platelet sensitivity to sensitizer BH3 peptides to measure dependence on pro-survival proteins. Platelets from WT mice readily underwent MOMP (indicated by cytochrome c release) in response to BAD (inhibits BCL-2, BCL-X_L_ and BCL-W) and HRK (inhibits only BCL-X_L_) peptides (Fig. 3D), indicating that platelets are highly dependent on BCL-X_L_. WT platelets were also largely insensitive to the MS-1 peptide (Fig. 3D), which only inhibits MCL-1, demonstrating lack of dependence on MCL-1. Platelets from *Asgr2*^–/–^ mice showed increased cytochrome c loss relative to WT platelets in response to BAD, HRK and MS-1 peptides, demonstrating that older, desialylated platelets are more dependent on BCL-X_L_ and can even trigger apoptosis when MCL-1 is inhibited due to the extremely high priming status of these cells. Conversely, *St3gal4*^–/–^ platelets, which lack sialylation but are young, were much less sensitive to the HRK peptide, indicating reduced BCL-X_L_ dependence (Fig. 3D). These results suggest that both platelet aging and sialylation status impacts BCL-X_L_ dependence.

We next sought to determine if platelet sialylation status can modulate BCL-X_L_ dependence independently of platelet age. WT mice were treated with the sialidase inhibitor DANA (2,3-dehydro-2-deoxy-N-acetylneuraminic acid) to prevent platelet desialylation in vivo [13]. Platelets from DANA-treated animals were less sensitive to the BID activator and PUMA sensitizer peptides when compared to WT platelets, indicating these platelets are less primed for apoptosis (Fig. 3D). Further, DANA treatment also resulted in reduced BAD sensitivity in platelets compared to WT, demonstrating that maintaining sialylation in circulating platelets reduces BCL-X_L_ dependence.

### Diverse BH3 mimetics targeting BCL-X_L_ selectively induce apoptosis in human and mouse platelets

**Fig. 4:**
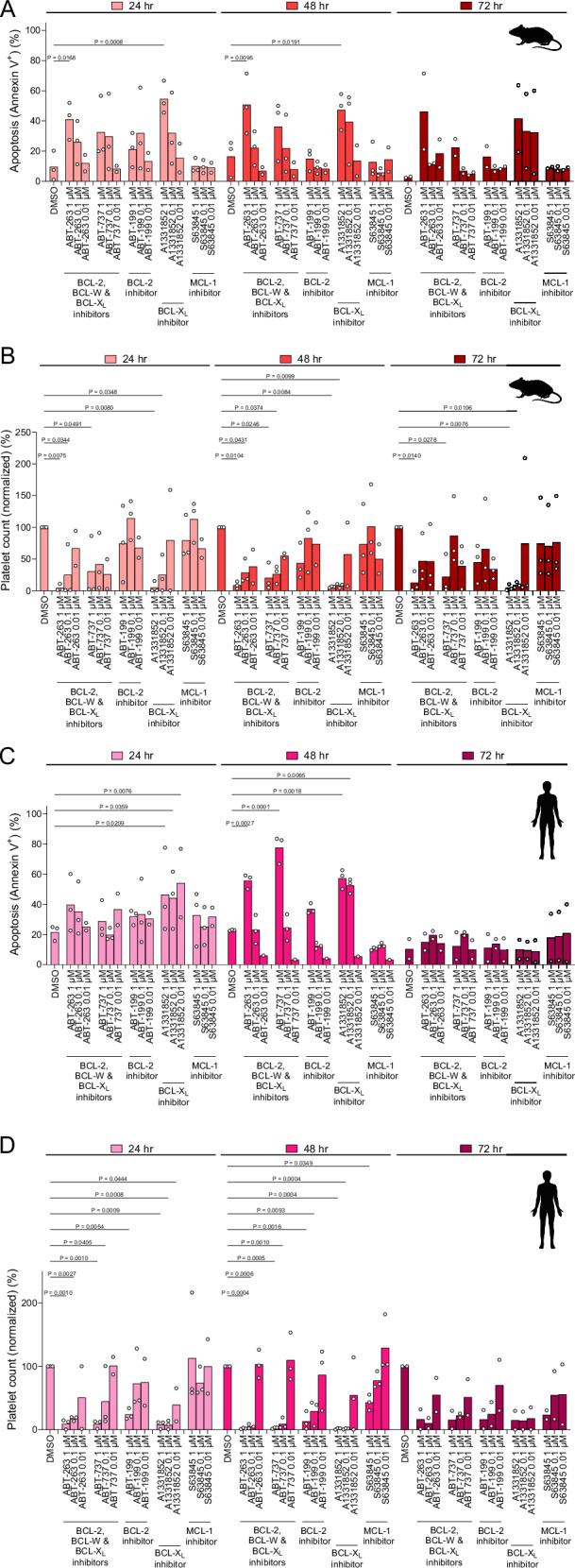
Murine and human platelets undergo apoptosis in response to BCL-X_L_ inhibitors. Flow cytometry analysis of **A**, **B** WT murine or **C**, **D** human platelets treated with BH3 mimetics in vitro for indicated periods using **A**, **C** Annexin V to detect apoptosis or **B**, **D** platelet counts. Mean is shown for *n* = 2–3 biological replicates, as indicated by individual data points.

Since mouse and human platelets vary in their expression of BCL-2 family proteins and have been shown to express BCL-2 [45] and MCL-1 [46], we sought to compare their sensitivity to BH3 mimetics by targeting all major pro-survival proteins. We found that mouse platelets rapidly (within 24 h) undergo apoptosis in response to the BCL-2, BCL-W and BCL-X_L_ inhibitors ABT-263 (navitoclax) and ABT-737 at 1.0 μM dose and exhibited even higher levels of apoptosis in response to the BCL-X_L_ inhibitor A1331852 in vitro (Fig. 4A). Interestingly, Annexin V-positive platelets continued to be detected at 48 and 72 h after initial treatment with these agents. No significant Annexin V positivity was detected on mouse platelets treated with the BCL-2 inhibitor ABT-199 (venetoclax) or the MCL-1 inhibitor S63845. It’s important to note that although S63845 has six-fold higher affinity for human MCL-1 as compared with mouse MCL-1, it does inhibit the murine protein efficiently [47]. In addition to Annexin V positivity, we also counted platelets due to their potential rapid degradation in vitro, which may impair their detection via Annexin V staining. Mouse platelet numbers decreased dramatically in response to 1.0 and 0.1 μM doses of ABT-263 or ABT-737 across all time points (Fig. 4B). Treatment with A1331852, which specifically inhibits BCL-X_L_, also consistently reduced platelet counts at 1.0 and 0.1 μM doses across all time points. Interestingly, a mild but not significant decrease in platelet counts was observed after 24, 48 and 72 h treatment with ABT-199 while S63845 had no effect (Fig. 4B).

Human platelet data largely mirrored mouse results. We detected high levels of apoptosis induction by all BCL-X_L_ inhibitors (Fig. 4C). In addition, the 1.0 μM dose of ABT-199 induced significant apoptosis in platelets, especially at the 48 h time point. The MCL-1 inhibitor had no effect. Human platelet counts were extremely rapidly reduced by BCL-X_L_ inhibitors, even at the 0.1 μM doses (Fig. 4D). ABT-199 again had a discernable effect and reduced platelet counts at the highest dose tested while the MCL-1 inhibitor did not affect platelet counts. Overall, our data demonstrate similar sensitivity to BH3 mimetics between mouse and human platelets and very high levels of platelet sensitivity to inhibitors of BCL-X_L_. Our data also uncovered a mild yet consistent platelet sensitivity to ABT-199.

### Ex vivo DANA treatment blocks desialylation of platelets, reducing sensitivity to BCL-X_L_ inhibitors

**Fig. 5:**
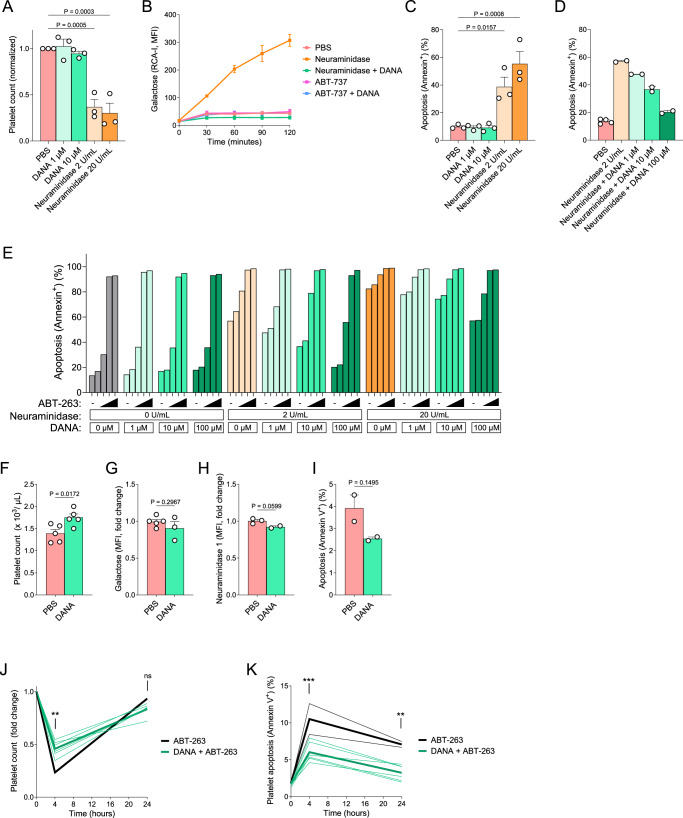
Desialylation directly induces apoptosis in platelets ex vivo and can be inhibited by DANA treatment. **A** Flow cytometry analysis of freshly isolated human platelets after ex vivo treatment with 2,3-dehydro-2-deoxy-N-acetylneuraminic acid (DANA) or neuraminidase for 24 h. Mean ± SEM is shown for *n* = 3 biological replicates, as indicated by individual data points. **B** RCA-I staining to detect exposed galactose in freshly isolated human platelets after ex vivo treatment with DANA, neuraminidase, ABT-263 or a combination of agents for up to 120 min. Mean ± StDev is shown for *n* = 2 biological replicates. Annexin V staining to detect apoptosis induced by **C** single agent treatments or **D**, **E** combination treatments with indicated agents for 24 h. Mean ± SEM is shown for *n* = 3 biological replicates in (**C**), mean ± StDev is shown for *n* = 2–4 biological replicates in (**D**) and mean is shown in (**E**) for *n* = 1–2 biological replicates. Flow cytometry analysis of platelets collected from WT mice treated with DANA or saline intraperitoneally every 12 h for 48 h total using **F** platelet counts, **G** RCA-I to detect exposed galactose, **H** anti-Neu1 antibody to detect surface expression of Neu1, and **I** Annexin V to detect apoptosis. Mean ± SEM is shown for *n* = 2–5 biological replicates, as indicated by individual data points. Platelet count (**J**) or platelet Annexin V positivity (**K**) from WT mice 4 or 12 h after a single intraperitoneal injection of ABT-263 (100 mg/kg) with or without DANA pretreatment. Data collected from each mouse as well as mean is shown for *n* = 2 (ABT-263) or 5 (DANA + ABT-263) mice/group.

Having observed that desialylated platelets are more primed to undergo apoptosis and more sensitive to BCL-X_L_ inhibition, we then asked if reducing platelet desialylation by treating platelets with DANA could prolong their survival. To test this, platelets were freshly isolated from human blood and treated in vitro with neuraminidase to induce desialylation or with DANA to prevent desialylation. We first counted platelets 24 h after treatment and found that neuraminidase significantly reduced platelet numbers, which was consistent with mouse platelet data (Fig. 2H), whereas DANA treatment had no effect on platelet counts (Fig. 5A). Neuraminidase-treated platelets were desialylated in vitro (Fig. 5B) and underwent apoptosis at significantly higher rates when compared to DANA-treated or untreated platelets, as measured by Annexin V positivity (Fig. 5C). We then tested if DANA treatment could restrict neuraminidase-induced desialylation in platelets. We found that pre-treatment with DANA indeed eliminates neuraminidase-mediated desialylation of human platelets (Fig. 5B, D) and reduced platelet apoptosis in a dose-dependent manner, with 100 μM DANA treatment resulting in near undetectable levels of Annexin V positivity (Fig. 5D).

We then sought to test the extent to which DANA treatment affects BCL-X_L_ dependence in neuraminidase-treated platelets. We observed that human platelets were highly sensitive to ABT-263 (navitoclax) and neuraminidase treatment further enhanced this sensitivity (Fig. 5E). Significantly, DANA treatment eliminated the ABT-263 sensitization produced by neuraminidase at 2 μ/mL and reduced sensitization even at 20 μ/mL (Fig. 5E), while not affecting ABT-263-induced apoptosis in the absence of neuraminidase. These data indicate that reduction of neuraminidase-induced platelet desialylation by DANA treatment reduces the sensitivity of platelets to BCL-X_L_ inhibitors.

### DANA treatment prevents desialylation in vivo, prolonging platelet survival

To assess whether endogenous desialylation-induced platelet apoptosis can be inhibited in vivo, WT mice were treated with PBS or DANA over a 48-h period. We found that DANA treatment led to an increase in overall platelet counts (Fig. 5F). Consistent with in vitro data, DANA treatment seemed to reduce desialylation of aging platelets in vivo as determined by RCA-I binding and neuraminidase 1 surface exposure (Fig. 5G, H) and also reduce ongoing platelet apoptosis (Fig. 5I), but these differences were not statistically significant.

Having previously observed that DANA-treated mouse platelets were less dependent on BCL-X_L_ for survival by BH3 profiling (Fig. 3D), we then asked if in vivo DANA treatment could protect platelets from BCL-X_L_ inhibitors. To test this, WT mice were treated with PBS or DANA for 48 h and then received one injection of ABT-263 followed by blood collection at 4 and 24 h. DANA-treated mice exhibited improved platelet counts (–55%) after ABT-263 administration compared to PBS-treated animals (–77%) 4 h after treatment (Fig. 5J). Platelet levels returned to normal after 24 h in both sets of animals (Fig. 5J) but required higher platelet production in the absence of DANA treatment. We also detected significantly less apoptotic platelets in DANA-treated animals (Fig. 5K). Overall, these data demonstrate that DANA treatment reduces platelet desialylation and renders them more resistant to apoptosis induced by BH3 mimetics that inhibit BCL-X_L_.

### Young human platelets are less primed for apoptosis, less dependent on pro-survival BCL-X_L_

**Fig. 6:**
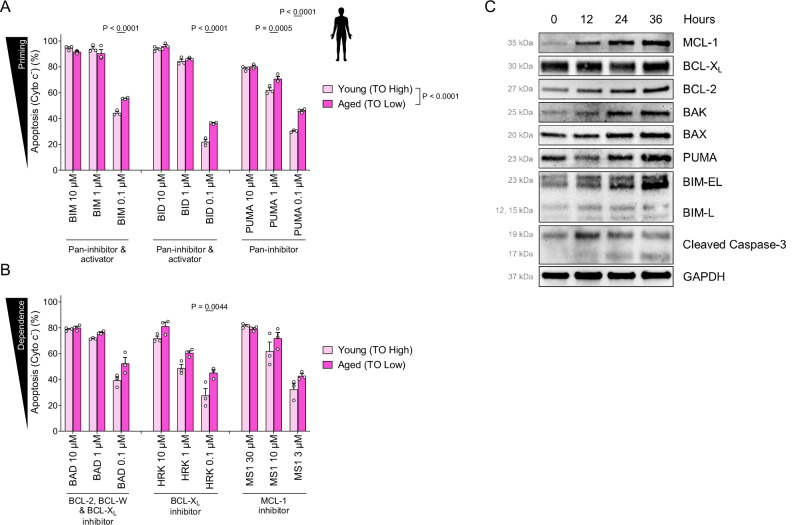
Young human platelets are less primed for apoptosis and less dependent on BCL-X_L_ for survival. BH3 profiling of freshly isolated young (thiazole-orange^high^) and aged (thiazole-orange^low^) human platelets to measure apoptotic priming (**A**) or dependencies (**B**). Mean ± SEM is shown for *n* = 3 biological replicates, as indicated by individual data points. **C** Freshly isolated human platelets were incubated at room temperature for indicated time periods and subjected to immunoblotting with antibodies recognizing indicated protein with GAPDH used as loading control. Data are representative of *n* = 3 biological replicates.

We next sought to determine how platelet aging in vivo affects apoptosis sensitivity. To test this in human cells, we stained freshly collected platelets with thiazole orange and subjected them to BH3 profiling. Thiazole orange allows for measurement of nucleic acid content in platelets [48], which decreases during their lifespan in circulation [49]. Older (thiazole orange low) human platelets containing lower levels of nucleic acids were more sensitive to apoptosis-promoting BIM, BID and PUMA BH3 peptides (Figs. 6A and S2A), indicating that they were more primed for apoptosis. Notably, the older platelets were also more sensitive to the BAD and HRK BH3 peptides that inhibit BCL-X_L_ and more sensitive to the MCL-1 inhibiting peptide MS1 (Fig. 6B). This indicated that aged human platelets had increased overall apoptotic priming and increased dependence on BCL-X_L_ compared to young platelets.

To uncover potential mechanisms driving this difference, we next measured expression of several key BCL-2 family proteins in freshly-collected human platelets that were aged in vitro for 12, 24, and 36 h. We found that expression of multiple pro-apoptotic proteins increases consistently during in vitro aging, including BIM, BAK and PUMA (Fig. 6C). Balancing this increased pro-apoptotic protein expression, we also found that levels of pro-survival MCL-1 and BCL-2 also increase, while BCL-X_L_ levels remain consistent. The net result of these changes would be increased pro-apoptotic proteins being sequestered by the pro-survival proteins, leading to the heightened apoptotic priming and BCL-X_L_ dependence that we detected via BH3 profiling in aging platelets. These changes were also associated with accumulating levels of cleaved caspase 3, which is consistent with the increasing apoptotic priming and ongoing apoptosis of these aged platelets.

### Young mouse platelets are less primed and less dependent on BCL-X_L_

**Fig. 7:**
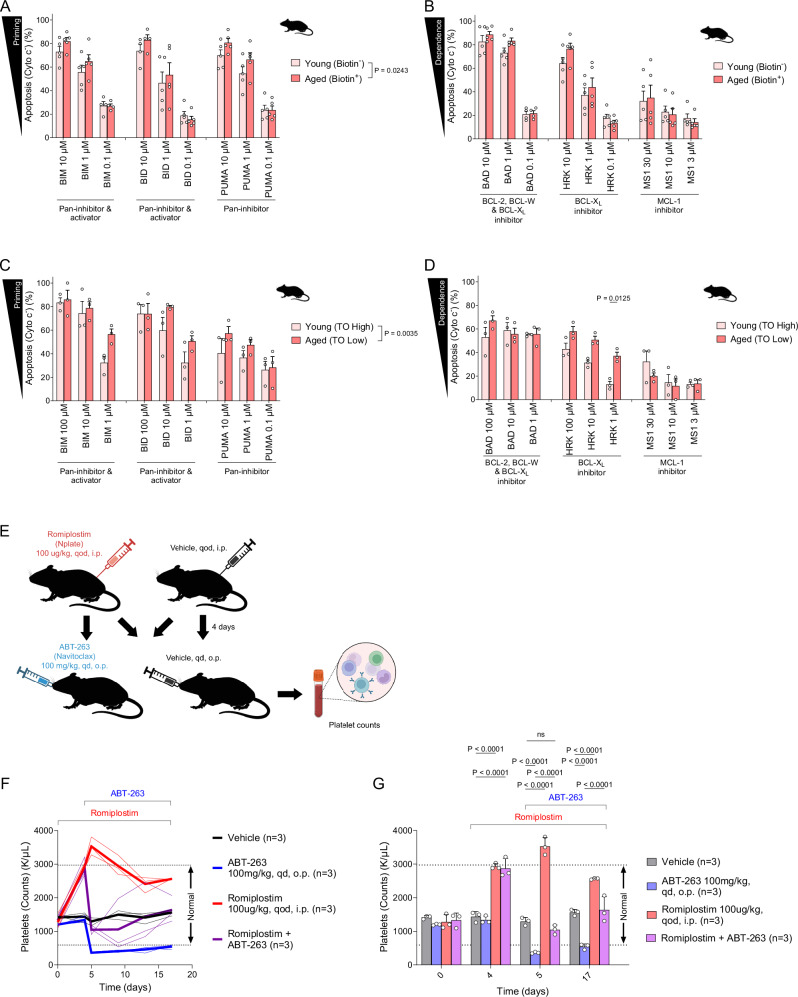
Young platelets are less primed for apoptosis, less dependent on BCL-X_L_; romiplostim prevents BCL-X_L_ inhibitor-induced thrombocytopenia. BH3 profiling of freshly isolated young (biotin^−^) and aged (biotin^+^) (platelets from WT mice injected with biotin to measure apoptotic priming (**A**) or dependencies (**B**) in vivo. Mean ± SEM is shown for *n* = 5 biological replicates, as indicated by individual data points. BH3 profiling of freshly isolated young (thiazole-orange^high^) and aged (thiazole-orange^low^) platelets from WT mice to measure apoptotic priming (**C**) or dependencies (**D**) in vivo. Mean ± SEM is shown for *n* = 3 biological replicates, as indicated by individual data points. **E** Workflow of experiment evaluating effects of romiplostim and ABT-263 on platelet counts. Created with BioRender.com. **F**, **G** Platelet counts in WT animals treated with vehicle, romiplostim (day 0 to 17), ABT-263 (Day 4 to 17) or combination for indicated time periods in vivo. Data collected from each mouse as well as mean is shown for *n* = 3 mice/group in (**F**) while mean ± SEM is shown for *n* = 3 mice/group at indicated number of days post initiation of treatments in (**G**).

Using the technique of in vivo biotinylation of WT mice, we next performed BH3 profiling of young versus aged platelets to compare their apoptotic priming and dependencies. Similar to human platelets, we found that aged (biotin^+^) platelets were more primed for apoptosis than young (biotin^−^) platelets based on sensitivity to the BIM, BID and PUMA BH3 peptides (Fig. 7A). In addition, the aged platelets also tended to exhibit increased sensitivity to the BCL-X_L_ inhibiting BH3 peptides including BAD and HRK, suggesting increased dependence on BCL-X_L_, although this difference was not statistically significant (Fig. 7B).

We next compared young versus aged platelets using thiazole orange staining. We again detected increased priming in aged (thiazole orange low) platelets relative to their young counterparts (Fig. 7C). We also detected increased BCL-X_L_ dependence based on higher cytochrome c release in response to the HRK BH3 peptide in the aged platelets (Fig. 7D). We also performed similar experiments comparing platelets from WT and *Bax*^*–/–*^ mice to measure the effect of BAX knockout on platelet priming and dependencies. BH3 profiling of platelets isolated from *Bax*^*–/–*^ mice showed that their priming levels were not significantly different (Fig. S2B, C). This is consistent with previous reports showing that knockout of *Bak1* (encoding BAK) causes a significant platelet accumulation while knockout of *Bax* does not [50]. Taken together, our data indicates that young platelets are less primed and less BCL-X_L_ dependent than aged platelets in both mice and humans.

### Thrombopoietin stimulates platelet production, prevents BH3 mimetic-induced thrombocytopenia

Based on our BH3 profiling results, we hypothesized that stimulation of new platelet production would lead to an increase in young platelets in circulation, which would be less sensitive to BCL-X_L_ inhibition due to their lower apoptotic priming and lower expression of pro-apoptotic proteins. We therefore treated mice with the thrombopoietin (TPO) receptor agonist romiplostim [51] for 4 days and detected a three-fold increase in circulating platelets compared with vehicle controls (Fig. 7E–G). We then treated animals with the BCL-2/BCL-w/BCL-X_L_ inhibitor ABT-263 at doses we and others have shown to be effective in cancer models [52, 53]. ABT-263 caused a rapid decrease in circulating platelets, which were below normal levels (692 K platelets per μL) within 1 day in vehicle-treated mice and remained abnormally low for the duration of the 14-day ABT-263 treatment. Strikingly, romiplostim treated animals also exhibited a decrease in platelet counts upon ABT-263 treatment but the nadir was, on average, over two-fold higher than in animals treated with ABT-263 alone and never reached abnormally low levels. We also observed a further recovery and normalization of platelet levels in the combination-treated mice, suggesting that sustained stimulation of platelet production can ameliorate the risk of thrombocytopenia from continuous BCL-X_L_ inhibition.

## Discussion

The recent development and clinical success of the BCL-2 inhibitor venetoclax (ABT-199) as treatment for chronic lymphocytic leukemia [54], acute myeloid leukemia [55] and other hematologic malignancies has shown that targeting apoptosis directly has the potential to significantly improve patient outcomes. Other BH3 mimetics targeting MCL-1 and BCL-X_L_ are also in various stages of preclinical and clinical development and have been shown to have activity in a myriad of cancer types including highly chemoresistant solid tumors [56, 57]. However, although highly potent BCL-X_L_ inhibitors such as ABT-737, ABT-263, A1331852 and others are available, one major impediment to their clinical use is the on-target and dose-limiting thrombocytopenia that these inhibitors cause in patients [23, 58]. Our studies suggest that two strategies may reduce platelet BCL-X_L_ dependence and prevent the thrombocytopenia that commonly occurs in patients treated with BH3 mimetics targeting this protein. First, we find that the neuraminidase (sialidase) inhibitor DANA is able to prevent platelet desialylation in vitro and in vivo and reduce sensitivity to BCL-X_L_ inhibition. Although this agent is not FDA approved, its analogue Zanamivir (4-GU-DANA), designed to inhibit viral neuraminidases, is FDA approved and used to treat and prevent influenza caused by influenza A and influenza B viruses [59]. Another potentially promising strategy would be to stimulate the thrombopoietin pathway in patients prior to treatment with BCL-X_L_ inhibitors—this would result in the production and release of young, sialylated platelets, which we found to be more resistant to BCL-X_L_ inhibition. Romiplostim, a fusion protein analog of thrombopoietin [51] and eltrombopag, a thrombopoietin receptor agonist [60], are both FDA approved for the treatment or prevention of various platelet disorders and may be effective in this role. Indeed, we found that romiplostim pre-treatment for 4 days prior to the daily administration of the BCL-2 and BCL-X_L_ inhibitor ABT-263 raised platelet production and prevented treatment-induced thrombocytopenia.

Recently, novel BCL-X_L_ proteolysis targeting chimeras (PROTACs) have been developed to degrade BCL-X_L_ in cancer cells but not platelets, as it targets BCL-X_L_ to the VHL E3 ligase for ubiquitination and proteasomal degradation and platelets express very low levels of VHL [61, 62]. Although these agents have been reported to be effective against multiple cancers while sparing platelets in preclinical studies [62], their clinical fate is yet unknown. Since platelet apoptosis stimulates the thrombopoietin pathway, others have suggested that ramp-up dosing of BCL-X_L_ inhibitors may help stimulate endogenous TPO production and avoid severe thrombocytopenia that can be life threatening [63]. However, the rapid adaptation of cancer cells to sub-lethal doses of BH3 mimetics may actually facilitate the development of therapy resistance [62, 64], suggesting that ramp-up dosing may reduce the long-term efficacy of these agents. Stimulation of platelet production with romiplostim or other agents with similar mechanisms of action is an attractive alternative strategy to prevent severe thrombocytopenia with non-degradative BCL-X_L_ inhibitors.

Our studies also uncovered a mild yet consistent platelet toxicity induced by ABT-199 (venetoclax). About 15% of patients treated with venetoclax experience drug-induced thrombocytopenia [65], which is likely to be linked to the sensitivity reported here. As with BCL-X_L_ inhibitors, the use of strategies that can preserve sialylation of circulating platelets or stimulate release of younger and less primed platelets may prevent this potentially dose-limiting toxicity.

Our work also uncovered novel aspects of apoptosis regulation in platelets. We found that expression of pro-apoptotic proteins including BIM, BAK and PUMA increase with time when platelets are cultured in vitro as a model of platelet aging. Concurrently, expression of pro-survival proteins including MCL-1 and BCL-2 also increase while high expression of BCL-X_L_ is consistently maintained. The net functional effect of these protein changes in aged platelets is a push toward apoptosis, as evidenced by their higher apoptotic priming levels. Based on this, the reduced sensitivity to BCL-X_L_ inhibition in young platelets likely stems from decreased overall expression of pro-apoptotic proteins as opposed to increased expression of other pro-survival proteins.

The accumulation of pro-apoptotic and pro-survival protein expression in anucleate platelets that lack the ability to transcribe new mRNAs is notable, especially since MCL-1 has a very short protein half-life in other cell types [66]. With degradative pathways being altered in platelets to help regulate their activation and other functions [67], it is possible that stability of BCL-2 family proteins is regulated in a unique manner in platelets. Interestingly, we did not observe any platelet cell death in response to MCL-1 inhibition in vitro (Fig. 4A–D), suggesting that although MCL-1 can be expressed in platelets, BCL-X_L_ plays a more dominant role in maintaining platelet survival. This is consistent with our detection of only mild sensitivity to the MCL-1 inhibiting MS1 peptide in platelet BH3 profiling experiments.

Our findings provide the first evidence of a clear link between platelet desialylation and the induction of apoptosis, which were previously thought to be independent yet complementary regulators of platelet lifespan in vivo (see schematic Fig. S3). We found that direct activation of mitochondrial apoptosis in platelets did not result in platelet desialylation; however, endogenous (in vivo) or pharmacologic (in vitro) desialylation of platelets induced apoptosis. Although the mechanisms that are responsible for increasing apoptotic priming in platelets that have been desialylated are unclear, there have been multiple studies implicating certain sialic acid residues as promoters of survival signaling in platelets. For example, N-acetyl neuramininic acid (NANA) helps support proper platelet aggregation, adhesion and contributes to platelet’s electrophoretic mobility [68]—the loss of any of these normal platelet functions could eliminate pro-survival signaling and trigger apoptosis. Future work will focus on identifying the functions that directly promote survival signaling in platelets. Interestingly, platelets isolated from mice deficient in sialylation (*St3gal4*^–/–^) were not sialylated and underwent apoptosis more readily in vivo but actually were less primed for apoptosis. This difference likely stems from the more rapid turnover of platelets in these mice— the cleaved caspase 7 staining (Fig. 1F) indicates they have a higher rate of caspase 7 activation at baseline and would be rapidly cleared, which would chronically stimulate further platelet production. Indeed, more rapid apoptosis, lower platelet counts and increased platelet size (indicative of more recent production by megakaryocytes) were all previously reported in *Stgal4*^–/–^ mice [69]. Based on this, the platelets that we analyzed via BH3 profiling most accurately reflect “young” platelets that were recently released into circulation, thus contributing to their low level of priming that is similar to young platelets collected in WT mice. In this case, the increased apoptotic stress triggered by lack of platelet sialylation in the *Stgal4*^*–/–*^ platelets is counterbalanced by the reduced apoptotic priming of young platelets, resulting in apoptotic priming measurements similar or even a slightly lower than WT platelets. It is also possible that the process of desialylation produces apoptotic stress, not the lack of sialic acid residues itself. Further research is needed to help clarify this relationship.

Our work has clinical implications beyond the use of BCL-X_L_ inhibitors. Based on our findings, prevention of platelet desialylation with sialidase inhibitors such as DANA, or more specific inhibitors that are available, can prolong the survival of existing platelets in circulation and enhance platelet counts. This effect may potentially be leveraged as treatment for platelet disorders such as chronic idiopathic (immune) thrombocytopenic purpura (ITP), where patients with abnormally low platelet levels are treated with thrombopoietin agonist to promote platelet production and strive to overwhelm the ongoing immune-mediated destruction of platelets [70] as well as for cancer patients that frequently experience thrombocytopenia resulting from cytotoxic chemotherapy treatment [8].

## Methods

### Ethics approval and consent to participate

This project and all described experiments were conducted in accordance with the guidelines and regulations of the Committee on Microbiological Safety at the Harvard T.H. Chan School of Public Health under approved COMS Protocol # 17-245 and complies with all ethical and safety regulations mandated by said institution. Animal studies were conducted in accordance with the guidelines and regulations of the Institutional Animal Care and Use Committee (IACUC) at the Harvard T.H. Chan School of Public Health under approved IACUC Protocol #IS00001059. Blood collection from human donors was conducted in accordance with the guidelines and regulations of the Institutional Review Board at the Harvard T.H. Chan School of Public Health under approved IRB Protocol # IRB23-1756 after obtaining a written statement of informed consent.

### In vivo biotinylation of platelets and isolation of young and old murine platelets

WT mice received a single dose of NHS-biotin (Calbiochem) intravenously and blood was collected 60 h after biotin injection. Platelets were purified as previously described [16]. Freshly isolated washed platelets (10^8^/ml) were incubated with anti-biotin microbeads (Miltenyi Biotech) at room temperature, according to manufacturer’s instructions. The platelet-microbeads mixture was subjected to LS columns (Miltenyi Biotech) for magnetic separation of labeled cells. The biotin negative fraction (young platelets) was collected within the first pass, and after careful washes, the column was removed from the magnetic stand and the biotin positive fraction (old platelets) was collected (see Fig. S1).

### Isolation of mouse platelets without biotinylation

Blood was collected via retroorbital bleed from male and female mice between 90 and 180 days of age of the following strains: C57BL/6J (WT) (The Jackson Laboratory Strain #:000664), B6.129-*St3gal4*^*tm1.1Jxm*^/J (*Stgal4*^*–/–*^) (The Jackson Laboratory Strain #:006900), B6;129S7-*Asgr2*^*tm1Her*^/J (*Asgr2*^*–/–*^*)* (The Jackson Laboratory Strain #:002361) in accordance with the guidelines and regulations of the Institutional Animal Care and Use Committee (IACUC) at the Harvard T.H. Chan School of Public Health, under approved protocol #IS00001059. Platelet isolation was performed as previously described [11]. Platelet counts, in all experiments, were determined by flow cytometry using 5.5 µm diameter SPHERO rainbow beads (Spherotech) as a reference [11].

### Isolation of human platelets

As previously described [11], blood was collected from human donors after obtaining informed consent under an IRB-approved protocol at Harvard University (Protocol # IRB23-1756) into lavender-top (EDTA-containing) tubes. Blood was centrifuged for 20 min at 177 × *g* at room temperature with low brake. The supernatant was collected and transferred to a new tube and prostaglandin E1 (100 μM PGE1, Sigma) was added to a final dilution of 1:10,000 of stock 10 mM solution. Solution was centrifuged for 5 min at 1000 × *g* at room temperature and pellet (containing platelets) was resuspended with HBSS and counted.

### Flow cytometry

To determine surface expression of specific glycan residues on platelet, freshly isolated washed platelets were stained with RCA-I FITC-conjugated lectin to quantify terminal galactose residues. All lectins are purchased and validated by Vector Laboratories. To determine and quantify surface expression of different platelet proteins, specific antibodies fluorescently-conjugated antibodies were used (AF488-NEU1, PE or FITC-P-selectin, APC or FITC-Annexin V),

### Western blot/immunoblot analysis

Freshly isolated platelets were lysed in 3× NP-40 lysis buffer (3% Nonidet-40, 150 mM Tris, 450 mM NaCl, 200 mM EGTA, 200 mM NaVO_3_) for 30 min in ice. Lysates were centrifuged, supernatants were collected. Protein concentration was measured by Pierce™ Microplate BCA Protein Assay Kit (Thermo Fisher Scientific). Samples were subjected to 4% to 12% SDS-PAGE gels and subsequently transferred to PVDF membranes. Membranes were first blocked with 5% milk and then incubated with primary antibodies overnight at 4 °C. After washing with PBST, they were incubated with the corresponding secondary antibodies for 2 h at room temperature. Subsequent washes were followed by incubation with SuperSignal West Femto Stable Peroxide and Luminol/Enhancer reagents (Thermo Fisher Scientific). Signal detection was performed using the ECL Detection System (Thermo Fisher Scientific). The antibodies used were as follows: actin—Sigma A2066; BAX—Cell Signaling 2772; BCL-X_L_—Cell Signaling 2764; full length and cleaved caspase 7—Cell Signaling 9491; GAPDH—Cell Signaling 5174; MCL-1—Cell Signaling 94296; BCL-2—Cell Signaling 3492; BAK NT– Millipore 06-536; PUMA—Cell Signaling 98672; BIM—Cell Signaling 2933; Cleaved Caspase-3—Cell Signaling 9664; Anti-rabbit IgG (H1L) HRP conjugate—Thermo, 31460.

### BH3 profiling

BH3 profiling was performed as previously described [32, 36]. Briefly, platelets were resuspended in Mannitol Experimental Buffer 2 (MEB2), which contains 10 mM HEPES (pH 7.5), 150 mM mannitol, 150 mM KCl, 1 mM EGTA, 1 mM EDTA, 0.1% BSA, and 5 mM succinate. The resulting cell suspension was distributed into pre-loaded plates containing the specified BH3 peptides and 0.001% digitonin, followed by a 60-min incubation at 28 °C. As cells undergo mitochondrial outer membrane permeabilization, they released cytochrome c, which is detected at the single cell level by staining for loss of cytochrome c. The pro-apoptotic peptides are either general pro-apoptotic signals, such as activators (BIM, BID and PUMA) or those that are specific inhibitors of pro-survival proteins (see Fig. 1A) including BAD, HRK and MS1. After 60-min incubation, cells were fixed by adding 8% paraformaldehyde at a 1:4 dilution (final concentration of 2%) for 15 min. This was followed by neutralization with N2 buffer (1.7 M Tris base, 1.25 M glycine, pH 9.1). Fixed cells were then incubated overnight at 4 °C with DAPI (1:1000, Abcam) and anti-Cytochrome c-Alexa Fluor 647 (1:2000, clone 6H2.B4, BioLegend) in intracellular staining buffer composed of 0.2% Tween-20 and 1% BSA. Cytochrome c release was assessed using an Attune NxT flow cytometer equipped with an autosampler (Thermo Fisher Scientific).

### ABT-263 and romiplostim administration

Animal studies were conducted using C57BL/6J wild-type mice (The Jackson Laboratory) in accordance with the guidelines and regulations of the Institutional Animal Care and Use Committee (IACUC) at the Harvard T.H. Chan School of Public Health, under approved protocol #IS00001059. For in vivo studies involving romiplostim, mice received doses of romiplostim (100 μg/kg) via intraperitoneal (IP) injection every other day and ABT-263 (100 mg/kg) via oral gavage every day. Whole blood samples were collected at indicated time points after initiation of romiplostim and ABT-263 treatment and used for downstream analyzes as described [50]. For all in vivo experiments, to perform well-powered tests of our hypotheses, we calculated sample sizes based on preliminary data collected from mice treated with agents of interest. Our power analysis determined that the planned group sizes provide at least 95% power to detect statistically significant differences (*p* = 0.05) in the endpoints of interest. Animals were assigned to experimental groups by random number generation and experimenters were blinded as to the treatments that the animals received.

### ABT-737 administration to induce apoptosis

For in vivo studies, mice received a single dose of ABT-737 (75 mg/kg) via intraperitoneal injection (ref [52]). Whole blood samples were collected at time 0, 1 and 2 h after ABT-737 injection. For in vitro studies, freshly isolated washed platelets (2 × 10^8^/ml) were treated with ABT-737 (5 μM) for 20 min at 37 °C. Apoptosis was confirmed by phosphatidylserine (PS) exposure measured by Annexin V binding by flow cytometry.

### In vivo sialidase treatment

For in vivo studies, mice received a single dose of 50mU α2–3,6,8 neuraminidase (sialidase) from *C. perfringens* (Sigma-Aldrich) intravenously via retro-orbital injections.

### In vitro neuraminidase (sialidase) treatment

To cleave sialic acid in vitro, freshly isolated washed platelets were incubated with 5mU/10^8^ platelets/mL of α2–3,6,8 neuraminidase (NA, sialidase) from *C. perfringens* (Sigma-Aldrich) at 37 °C for 15 min and desialylation of surface proteins confirmed by flow cytometry analysis using RCA-I lectin.

### Sialidase inhibitor treatment

DANA (2,3-dehydro-2-deoxy-N-acetylneuraminic acid, pan inhibitor, Calbiochem). For in vivo studies, DANA (50 mU) or PBS (control) was injected, i.p., every 12 h for 2 days. For in vitro studies, freshly isolated washed platelets (10^8^/mL) were incubated with DANA for 20 min at 37 °C [31].

### Statistical analysis

Significance was tested by student’s *t* test, One-Way or Two-Way ANOVA with Holm-Sidak’s correction for multiple hypothesis testing using GraphPad PRISM software. For each experiment, sample size was chosen based on the means and standard deviations of the different subgroups in pilot experiments.

## Supplementary information

Supplementary Material

Source Data

Reproducibility checklist

## Data Availability

The uncropped western blots are available as the file “available in the supplementary materials upon request from the corresponding author.
