# Supplementary material for: Platelet aging and desialylation increase apoptotic priming and BCL-XL dependence
Source: Cell Death Dis. 2026 Jan 8;17(1):13. doi: 10.1038/s41419-025-08205-8 (PMC12783235; doi:10.1038/s41419-025-08205-8)
Supplement: Supplementary file 1 — Supplementary Material [file 41419_2025_8205_MOESM1_ESM.pdf]

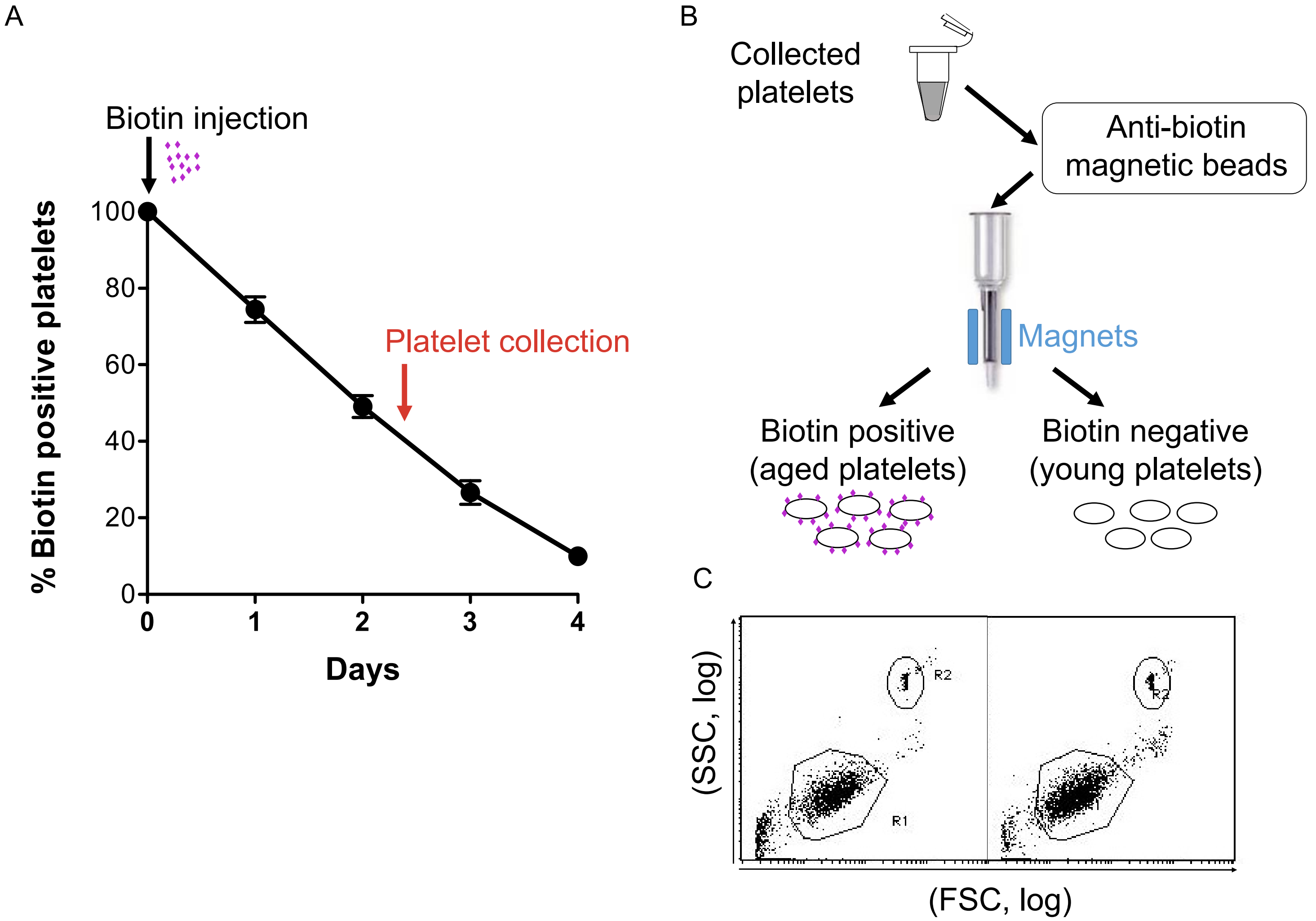


**Supplemental Figure 1: Scheme of *in vivo* biotinylation of WT platelets.** (A) Percentage of biotinylated circulating platelets following single intravenous injection of biotin in WT mice. The initial percentage of biotinylated platelets was set as 100%. Platelet half-life time point (~60 hr) is indicated. (B) Scheme of platelet separation into young (biotin$^{-}$) and aged (biotin$^{+}$) platelet populations. (C) Side-forward scatter of platelet populations after magnetic bead separation.

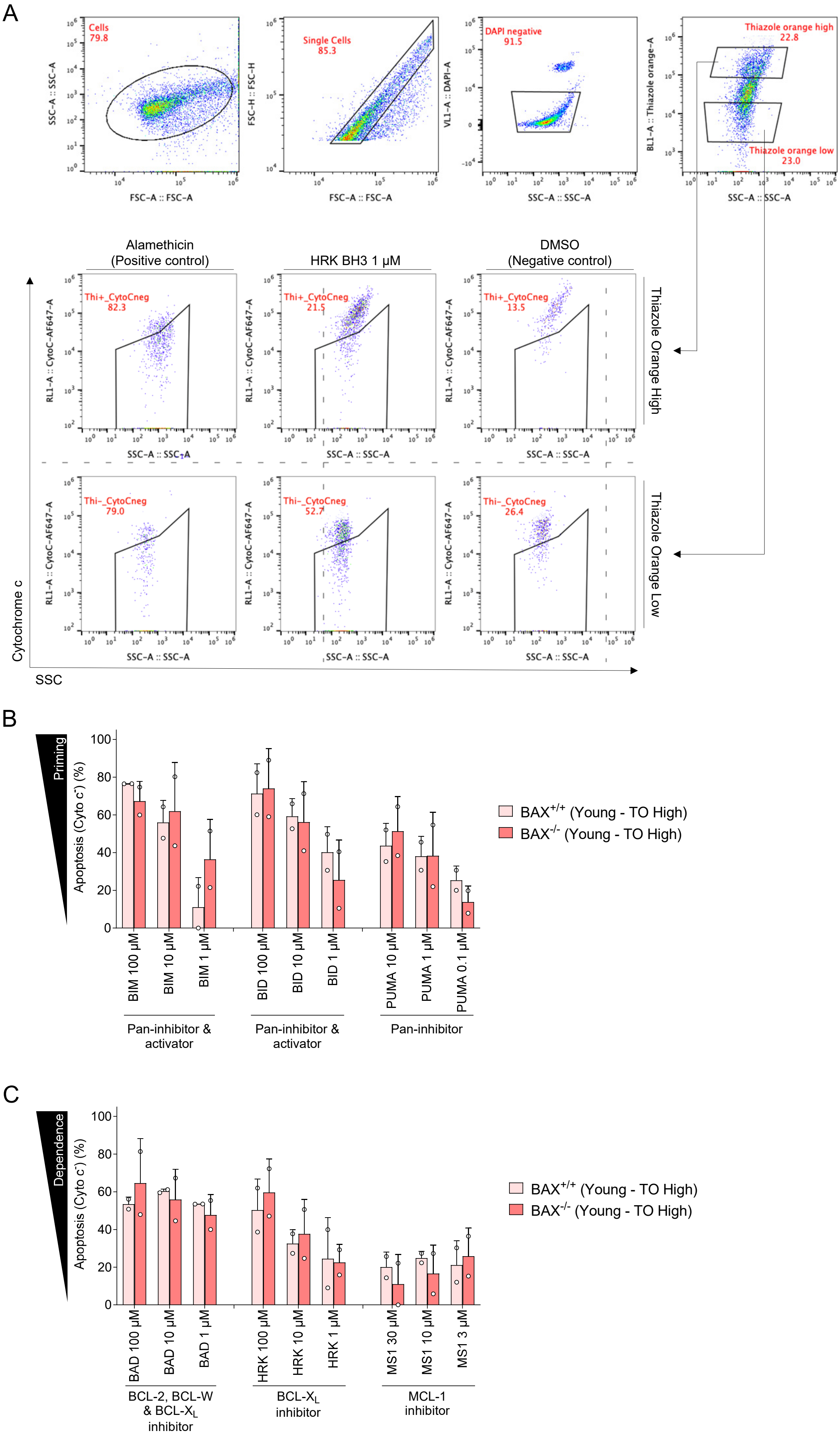


**Supplemental Figure 2: Apoptotic priming and dependencies in *Bax$^{-/-}$* platelets.** (A) Gating strategy for BH3 profiling of young versus aged mouse platelets based on thiazole orange staining. Shown is response to HRK BH3 peptide at 1 µM. (B-C) BH3 profiling of freshly isolated young (thiazole-orange$^{high}$) platelets from WT and *Bax$^{-/-}$* mice to measure apoptotic priming (B) or dependencies (C) in these genetic backgrounds. Mean ± StDev is shown for n = 2 biological replicates, as indicated by individual data points.

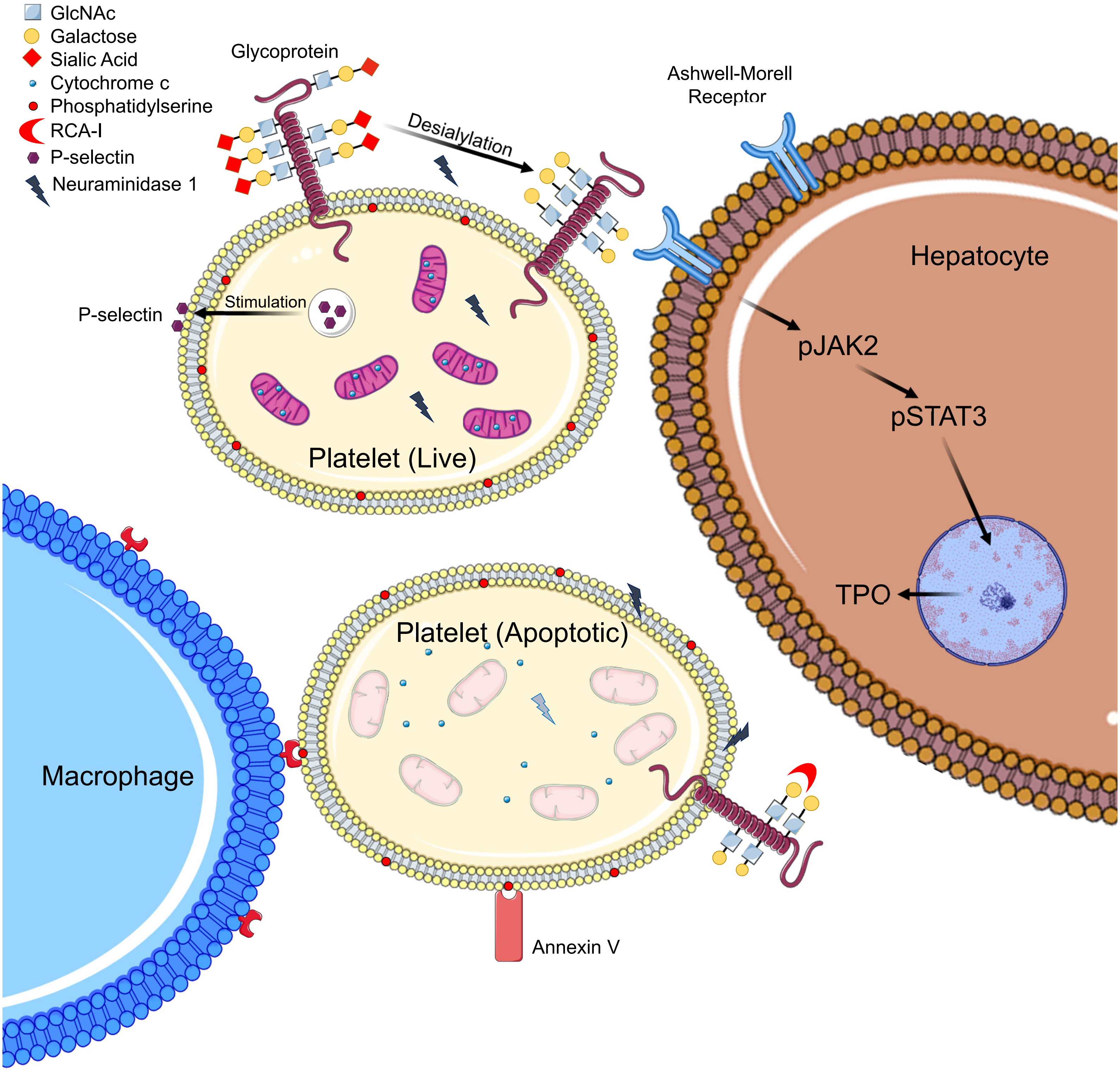


**Supplemental Figure 3: Proposed scheme of platelet aging process.** During circulation, neuraminidase present in the platelet cytoplasm gets transported to the surface where it removes sialic acid from surface glycoproteins and exposes galactose. The exposed galactose is recognized by the Ashwell-Morell Receptor on hepatocytes and triggers the internalization of the desialylated (aged) platelet. This process leads to the production of thrombopoietin to simulate further platelet production. In the absence of the AMR, platelets become more primed for apoptosis and then trigger this form of cell death.
